# Drug Induced Pneumonitis Secondary to Treatment with Paritaprevir/Ritonavir/Ombitasvir and Dasabuvir (VIEKIRA PAK®) for Chronic Hepatitis C: Case Report of an Unexpected Life-Threatening Adverse Reaction

**DOI:** 10.1155/2017/4895736

**Published:** 2017-03-20

**Authors:** Shih Yea Sylvia Wu, Bridget Faire, Edward Gane

**Affiliations:** Liver Transplant Unit, Auckland City Hospital, 2 Park Road, Grafton, Auckland 1023, New Zealand

## Abstract

VIEKIRA PAK (ritonavir-boosted paritaprevir/ombitasvir and dasabuvir) is an approved treatment for compensated patients with genotype 1 (GT1) chronic hepatitis C virus (HCV) infection. This oral regimen has minimal adverse effects and is well tolerated. Cure rates are 97% in patients infected with HCV GT 1a and 99% in those with HCV GT 1b. We report the first case of life-threatening allergic pneumonitis associated with VIEKIRA PAK. This unexpected serious adverse event occurred in a 68-year-old Chinese female with genotype 1b chronic hepatitis C and Child-Pugh A cirrhosis. One week into treatment with VIEKIRA PAK without ribavirin, she was admitted to hospital with respiratory distress and acute kidney injury requiring intensive care input. She was initially diagnosed with community acquired pneumonia and improved promptly with intravenous antibiotics and supported care. No bacterial or viral pathogens were cultured. Following complete recovery, she recommenced VIEKIRA PAK but represented 5 days later with more rapidly progressive respiratory failure, requiring intubation and ventilation, inotropic support, and haemodialysis. The final diagnosis was drug induced pneumonitis.

## 1. Introduction

The World Health Organization reports that 130–150 million people are infected with chronic hepatitis C infection (HCV) [1], of whom almost one million people die annually from either liver failure or liver cancer [2]. These numbers are expected to treble over the next 15 years [3]. Eradication of HCV infection through successful antiviral therapy will prevent liver-related complications [4]. Widespread access to effective therapies should prevent the projected global health burden and eventually eliminate HCV [5].

Patients infected with HCV GT1 have historically been the most difficult to cure with interferon-based therapies, where cure rates have been only half those achieved in patients with other HCV genotypes [6]. The introduction of direct antiviral agents (DAAs) has resulted in a paradigm shift in the management of patients with HCV infection [7]. VIEKIRA PAK comprises 3 DAAs targeting different HCV proteins—a NS3 protease inhibitor (ritonavir-boosted paritaprevir), a NS5A inhibitor (ombitasvir), and a NS5B polymerase inhibitor (dasabuvir). A 12-week course will cure more than 95% of patients [8–11], and this regimen has been approved by the European Medicines Agency (EMA) and US Food Drug Administration (FDA) for treatment of patients with HCV GT 1 infection.

VIEKIRA PAK is safe and well-tolerated treatment regimen with a well-established safety profile, with rare report of hepatotoxicity in patients with advanced cirrhosis [10]. We present the case of a Chinese woman who developed life-threatening allergic pneumonitis during VIEKIRA PAK therapy.

## 2. Case Presentation

A 68-year-old Chinese female was diagnosed with genotype 1b chronic hepatitis C in 2006 when she was noted to have mildly deranged liver function test with alanine aminotransferase (ALT) of 62. The likely source of HCV infection was blood transfusion in 1988 at the time of left mastectomy. Liver biopsy in 2007 showed mild fibrosis (Grade 3, Stage I). Her comorbidities included type 2 diabetes diagnosed in 1997 complicated by mild diabetic retinopathy, hypertension, dyslipidaemia, atrial fibrillation, laparoscopic cholecystectomy in 2013 for gallstone disease, and previous breast cancer cured in 1988.

Due to concerns regarding the safety and efficacy of interferon, she declined therapy and was followed annually in Hepatitis Clinic. She did not drink alcohol. A FibroScan in February 2016 demonstrated liver stiffness measurement of 12.4 KPa consistent with established cirrhosis. She had normal liver synthetic function and no evidence of portal hypertension. Her baseline blood tests included normal platelet of 230 ×  *E*9/L, haemoglobin 126 g/L, albumin 34 g/L, bilirubin 5 umol/L, and creatinine 75 umol/L (Table 1).

In view of the degree of liver disease she was offered treatment with VIEKIRA PAK through a national early access scheme. Potentially interacting medications including felodipine and atorvastatin were stopped, and she started 12 weeks of VIEKIRA PAK without ribavirin on 21 March. Her other medications including Penmix 30 50-unit mane, 40-unit nocte, NovoRapid 12 units at lunchtime, cholecalciferol, losartan, and dabigatran 110 mg bd were continued.

**Figure 1:**
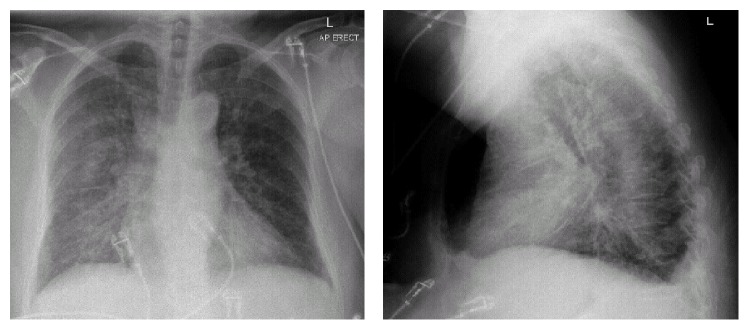
CXR on first admission.

**Figure 2:**
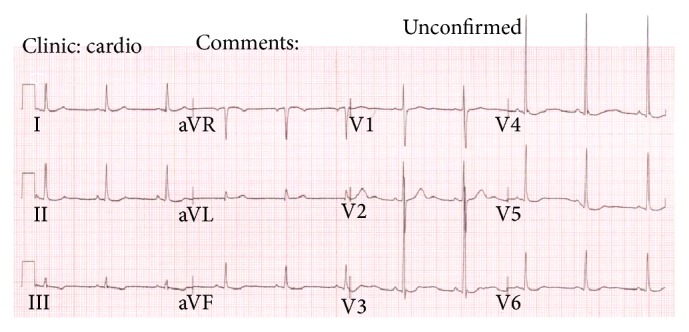
ECG on admission.

One week later, she presented acutely to hospital with a two-day history of fever, cough, headache, and palpitation and VIEKIRA PAK was suspended on admission. Her observations were temperature of 37.8°C, HR 128/min, BP 144/80, and oxygen saturation of 92% on 2 L of oxygen. Jugular venous pressure was not elevated, heart sounds were dual with no murmur, she had crepitations at the right lung base, and abdomen was soft and nontender without peripheral oedema. She had no evidence of meningism or petechiae. Chest X-ray showed pulmonary interstitial changes in the right lung without frank consolidation (Figure 1). ECG showed fast atrial fibrillation at a rate of 129/mins with T wave flattening in leads II, avF, V4, V5, and V6 (Figure 2). She was admitted to the ward with the provisional diagnosis of community acquired pneumonia and was started on intravenous cefuroxime and erythromycin. Due to worsening hypoxaemia, respiratory fatigue, tachycardia, and development of acute kidney injury she was admitted to the Critical Care Unit for intensive support. Over the next 24 hours, she rapidly improved and was transferred back to the respiratory ward. Echocardiogram (ECHO) on 30 March revealed normal left ventricular and right ventricular size and function, with only mild tricuspid regurgitation and trivial aortic regurgitation. She continued to improve. She was discharged after 5 days in hospital on a further 10-day course of oral amoxycillin and roxithromycin with the diagnosis of community acquired pneumonia.

**Figure 3:**
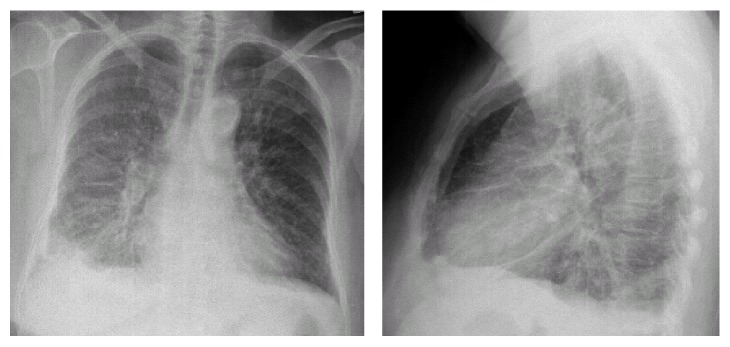
CXR on second admission.

**Figure 4:**
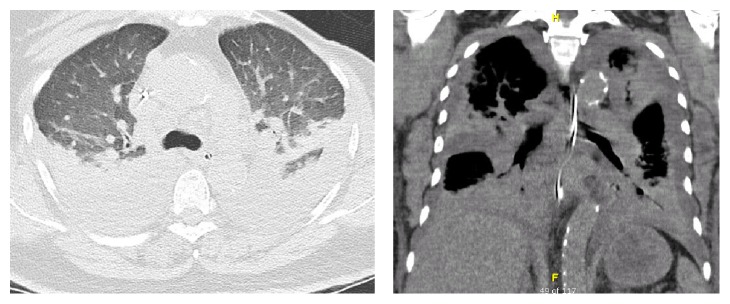
High resolution CT.

She was reviewed in outpatient clinic 3 weeks later. She was asymptomatic and returned to normal activities with no restrictions. Postdischarge blood tests were normal (Table 1). Given good recovery, she was restarted on VIEKERA PAK. She represented unwell with fever, cough, shortness of breath, headache, and back pain 4 days later. Her temperature was 38°C, heart rate was 104/mins, BP was 127/58, and oxygen saturation was 96% room air. Chest X-ray on admission showed bilateral pulmonary infiltrate more prominent on the right (Figure 3). Computed tomography (CT) noncontrast head scan excluded intracranial haemorrhage as a cause for headache. The respiratory service restarted empiric cefuroxime, roxithromycin, and oxygen support. She was in fast atrial fibrillation and required stat dose of 300 mg intravenous amiodarone to manage her heart rhythm. She continued to deteriorate rapidly. She became increasingly hypoxaemic and developed lactic acidosis and oliguria. She was again transferred to the Critical Care Unit for presumed systemic sepsis. She initially received continuous positive airway pressure (CPAP) but due to fatigue was intubated and ventilated. Despite fluid resuscitation and inotropic support, anuric renal failure ensued and she was commenced on Continuous Veno-Venous Haemodiafiltration (CVVHDF). A high resolution CT of the chest demonstrated widespread interstitial oedema, ground glass opacities, moderate bilateral pleural effusions with atelectasis of upper and lower lobes, and increased lung attenuation suggesting cellular infiltrate related pneumonitis (Figure 4). Repeat ECHO performed showed normal biventricular function with mild atrial regurgitation, moderate mitral regurgitation, and severe tricuspid regurgitation in the context of moderately dilated right ventricle. Autoimmune and vasculitis screen including antinuclear antibodies, antineutrophil cytoplasmic antibody (ANCA), anti-Sjögren's-syndrome-related antigen A (Anti-SSA/Ro), and rheumatoid factor were negative. Cryoglobulins and complement levels were not performed.

Reviewing results from her first admission, urinary legionella and pneumococcus were negative, and nasal pharyngeal viral polymerase chain reaction (PCR) panel and serial blood cultures were sterile. Repeat sputum and tracheal aspirates and pleural fluid aspirates had no evidence of bacterial, viral, or fungal infection. Given the similar presentations on rechallenging her with VIEKIRA PAK, a provisional diagnosis of drug induced interstitial pneumonitis was made and high dose corticosteroid therapy instituted. She received 500 mg intravenous methylprednisone daily for 4 days and clinical improvement was prompt. She was discharged home with normal gas exchange and renal function 7 days after admission. At the most recent follow-up nine months out from discharge, she remained clinically well with compensated liver disease.

## 3. Discussion

VIEKIRA PAK is a coformulated tablet containing paritaprevir/ritonavir/ombitasvir 75/50/12.5 mg copackaged with dasabuvir 250 mg. Paritaprevir inhibits the HCV NS3/4A protease and ombitasvir is an inhibitor of NS5A, both working to prevent viral replication. Dasabuvir is a nonnucleoside polymerase inhibitor. Ritonavir is a pharmacokinetic enhancer that increases the plasma drug concentration of paritaprevir without being active against HCV [12].

The AbbVie VIEKIRA PAK programme included seven randomised multicentre phase III trials involving at least 2,368 patients with genotype 1 HCV infection, including two dedicated studies in cirrhotic patients. Patients received VIEKIERA PAK with or without ribavirin for 12 or 24 weeks. Efficacy was excellent with >95% of patients achieving sustained virological response, including those with cirrhosis and those who had previously failed interferon-based therapy [8–11].

The safety of VIEKIRA PAK was also excellent with few treatment-related discontinuations—1.2% in patients who received additional ribavirin and only 0.3% in those who did not receive ribavirin. The common adverse reactions listed according to frequency were fatigue, nausea, pruritus, insomnia, asthenia, and anaemia. Other adverse events of less than 5% frequency include diarrhoea, vomiting, decreased haemoglobin, reduced appetite, dizziness, headache, cough, dyspnoea, dry skin, and rash. Adverse reactions in patients who received VIEKIRA PAK without ribavirin had safety profile similar to placebo. Transient, asymptomatic rise in ALT greater than five times the upper limits of normal was seen in 1% of patients. Hepatic decompensation and failure resulting in liver transplantation or fatal outcome have been reported in patients with advanced or decompensated liver disease prior to initiating VIEKIRA PAK. Hypersensitivity reactions including lip and tongue swelling have been documented in postmarketing experience [12].

This is the first reported case of drug induced pneumonitis associated with VIEKIRA PAK. Because the patient was infected with HCV genotype 1B, treatment did not include ribavirin. Although dabigatran coadministration has not been studied, exposure may theoretically be increased due to P-gp inhibition by VIEKIEA PAK. Her CHADsVASC score was 4 with an estimated annual stroke risk of 4%; therefore dabigatran was continued. On her first presentation given the haemoglobin drop from 100 g/L pretreatment to 88 g/L at the lowest, the differential included dabigatran induced pulmonary haemorrhage. Her presentations were not typical of pulmonary haemorrhage and she did not have haemoptysis. Although amiodarone-induced lung disease is well known, it was given as a 300 mg stat infusion after symptoms onset on her second presentation. It was unlikely the causative agent in her case. It is difficult to know which component of the VIEKIRA PAK caused the allergic reaction. Of the four drug components, ritonavir is an agent that has been utilised since 1996 after it was approved by FDA to treat Human Immunodeficiency Virus (HIV) infected patients. There has been no similar documented reaction in the literature in patients treated with regimen containing ritonavir.

Drug induced lung toxicity can affect the lung parenchyma, airways, pleura, pulmonary vasculature, mediastinum, and neuromuscular system, and the most common form is drug induced interstitial lung disease (DILD). Certain cytotoxic, anti-inflammatory agents and cardiovascular medications are known to cause drug related lung toxicity. Because the mechanism is not fully understood, the likelihood of adverse pulmonary effects from newly developed therapeutic medications remains unpredictable and largely idiosyncratic. Some risk factors identified are advanced age, female sex, genetic predisposition, and those with underlying lung disease. The pathogenesis of injury can include oxidative stress, immune-mediated or pulmonary vasculature damage, and direct toxic effect. DILD has no pathognomonic clinical, laboratory, physical, radiographic, or histologic findings. Patients usually present with fever, followed by respiratory symptoms such as dry cough, wheeze, and acute shortness of breath, and the onset can vary from days to even years. Symptoms from acute drug induced toxicity usually disappear 24–48 hours after the causative drug is withdrawn. In severe cases, DILD can result in respiratory failure requiring mechanical ventilation. Management involves prompt cessation of causative drug, supportive care, and consideration of corticosteroid. Mortality is associated with the degree of underlying fibrosis [13, 14]. In chronic drug exposure, respiratory symptoms may take longer to resolve. Lung disease progression despite drug discontinuation can be seen but may be related to long half-life of certain medications such as amiodarone [15].

Diagnosis of DILD is based on certain criteria. This includes a history of drug exposure, exclusion of other lung diseases, improvement following discontinuation of the suspected drug, and recurrence of symptoms on rechallenging with the same drug [16]. In this patient, the diagnosis of DILD became apparent when she developed the same clinical picture and imaging changes with repeat exposure to VIEKIRA PAK. Fortunately, she made full recovery from the respiratory compromise as well as the associated kidney injury after prompt cessation of the causative agents and corticosteroid administration.

In conclusion, VKEKIRA PAK plus ribavirin has been shown to have high efficacy for patients infected with genotype 1 chronic HCV without cirrhosis and with compensated cirrhosis. The adverse effects experienced in the seven randomised trials involving more than 2,000 patients were minimal and only led to discontinuation of treatment in very small number of people. Adverse reaction to such degree of severity requiring intensive care has not been previously reported since FDA approval and widespread use across the world, even in real-life cohort [17]. VIEKIRA PAK has been approved and funded in Australia since March 2016 and in New Zealand as of July 2016. As the number of patients treated with this combination increases, the real world data will elucidate the incidence of such reaction. Before starting patient on treatment, discussions about potential adverse effects, including rare but potentially serious idiosyncratic reaction, should be included in the consultation.

## Figures and Tables

**Table 1:** Baseline and subsequent laboratory results.

Laboratory results	Baseline	1st admission	Postdischarge	2nd admission	Follow-up
Haemoglobin (g/L)	126	100	111	102	113
Platelets (×*E*9/L)	230	350	145	304	290
Bilirubin (umol/L)	5	70	7	43	9
Albumin (g/L)	34	28	34	32	32
Creatinine (umol/L)	107	124	83	154	81
